# Gene polymorphisms and motor levodopa‐induced complications in Parkinson's disease

**DOI:** 10.1002/brb3.1537

**Published:** 2020-02-05

**Authors:** Małgorzata Michałowska, Małgorzata Chalimoniuk, Ewa Jówko, Iwona Przybylska, Józef Langfort, Beata Toczylowska, Anna Krygowska‐Wajs, Urszula Fiszer

**Affiliations:** ^1^ Department of Neurology and Epileptology Centre of Postgraduate Medical Education Orłowski Hospital Warsaw Poland; ^2^ Department of Physical Education and Health in Biała Podlaska Józef Piłsudski University of Physical Education in Warsaw Biała Podlaska Poland; ^3^ Institut of Sport Sciences Jerzy Kukuczka Academy of Physical Education in Katowice Katowice Poland; ^4^ Institute of Biocybernetics and Biomedical Engineering Polish Academy of Sciences Warsaw Poland; ^5^ Department of Neurology Jagiellonian University, Collegium Medicum Cracow Poland

**Keywords:** brain‐derived neurotrophic factor, catechol‐O‐methyltransferase, dopamine transporter, motor levodopa‐induced complications, Parkinson's disease, single‐nucleotide polymorphism

## Abstract

**Objective:**

The aim of the study was to evaluate the association of individual and combined single‐nucleotide polymorphisms in brain‐derived neurotrophic factor (BDNF), dopamine transporter (DAT), and catechol‐O‐methyltransferase (COMT) genes with the occurrence of motor levodopa‐induced complications (MLIC) in Parkinson's disease (PD).

**Materials and Methods:**

We studied 76 patients with PD (MLIC occurred in 56.6%) and 60 controls. Allelic discrimination of rs6265 BDNF (Val66Met), rs397595 DAT (*SLC6A3*), and rs4680 COMT (Val158Met) genes were genotyped. Odds ratios (OR) and 95% confidence intervals (95% CI) were calculated using multinominal logistic regression. Orthogonal partial least squares (OPLS) analysis and OPLS discriminant analysis (OPLS‐DA) were used to analyze qualitative genetic data.

**Results:**

The risk of PD in subjects with the AG BDNF genotype was increased sixfold (OR = 6.12, 95% CI = 2.88–13.02, *p* < .0001), and AG BDNF and AG DAT genotypes were correlated with PD in OPLS‐DA (VIP > 1). There were no differences in distributions of BDNF, DAT and COMT genotypes between PD groups with and without MLIC, while OPLS model showed that genotype combination of AG BDNF, AG DAT, and GG COMT was correlated with MLIC and genotypes combination of GG BDNF, AA DAT, and AA COMT with lack of MLIC in PD patients (VIP > 1).

**Conclusions:**

Our results confirmed the association of rs6265 BDNF (Val66Met) with the risk of PD and suggest a synergic effect of rs6265 BDNF (Val66Met), rs397595 DAT (*SLC6A3*), and rs4680 COMT (Val158Met) polymorphisms on the occurrence of MLIC.

## Graphical abstract

The aim of the study was to evaluate the association of individual and combined single‐nucleotide polymorphisms in brain‐derived neurotrophic factor (BDNF), dopamine transporter (DAT), and catechol‐O‐methyltransferase (COMT) genes with the occurrence of motor levodopa‐induced complications (MLIC) in Parkinson's disease (PD). Seventy‐six patients with PD and 60 controls were included. Allelic discrimination of rs6265 BDNF (Val66Met), rs397595 DAT (SLC6A3), and rs4680 COMT (Val158Met) genes were genotyped. The risk of PD in subjects with the AG BDNF genotype was increased sixfold, and AG BDNF and AG DAT genotypes were correlated with PD in orthogonal partial least squares discriminant analysis (OPLS‐DA). OPLS model showed that genotype combination AG BDNF, AG DAT, and GG COMT was correlated with MLIC and genotypes combination GG BDNF, AA DAT, and AA COMT with lack of MLIC in PD patients.

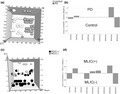

## 1 INTRODUCTION

Chronic dopaminergic therapy of Parkinson's disease (PD) is associated with a risk of motor levodopa‐induced complications (MLIC): motor fluctuations and dyskinesias. The inter‐individual heterogeneity in their development suggests a complex pathomechanism involving also genetic factors. Previous studies about genetic associations between MLIC and its pathophysiological mechanism reported however inconsistent results. The brain‐derived neurotrophic factor (BDNF) is thought to modulate synaptic transmission and plasticity and to mediate in neuronal survival, migration and differentiation in many regions of the brain, including on dopaminergic nigrostriatal neurons. Functional consequences of a common Val66Met single‐nucleotide polymorphism (SNP) of the BDNF gene (rs6265) involve decreased protein secretion in carriers of A (Met) allelic variants (Egan et al., 2003). That SNP has seemed to influence the dyskinesias onset in the course of PD treatment with dopaminergic agents (Foltynie et al., 2009). However, more recent studies did not evidence any association between several SNPs of the BDNF gene, including Val66Met, and the risk of developing levodopa‐induced dyskinesias (Cheshire et al., 2014; Kaplan et al., 2014). The dopamine transporter (DAT), a plasma membrane protein of dopaminergic neurons, plays an important role in controlling the intensity and duration of dopaminergic neurotransmission by rapid DA reuptake into presynaptic terminals. It was demonstrated that motor fluctuations in PD are associated with rapid increase in the synaptic levels of DA probably due to increased DA turnover related to lower DAT levels in nigrostriatal neurons (Fuente‐Fernández et al., 2001; Sossi et al., 2007). Two variations of the DAT gene, which functional effects are still unclear, seem to be correlated with levodopa‐induced dyskinesias in PD: the rs393795 SNP (Kaplan et al., 2014) and the 40‐base‐pair (pb) variable number tandem repeat (VNTR) polymorphism (Kaiser et al., 2003). Catechol‐O‐methyltransferase (COMT) and monoamine oxidases (MAO) are involved in the metabolism of amine neurotransmitters and play a central role in therapeutic response to levodopa. The allele A (Met) of a common SNP in the COMT gene, the Val158Met (rs4680), is linked to increased thermolability and reduced activity of the enzyme (Lotta et al., 1995). On the one hand, the susceptibility to MLIC was reported to be influenced by this polymorphism in COMT gene (Hao et al., 2014; de Lau, Verbaan, Marinus, Heutink, & Hilten, 2012; Watanabe et al., 2003; Wu et al., 2014), and on the other hand, no associations between COMT Val158Met and T941G MAO‐A SNPs either individually or combined and levodopa‐induced dyskinesias were found (Cheshire et al., 2014). The aim of the study was to evaluate the association of individual and combined SNPs in rs6265 BDNF (Val66Met), rs397595 DAT (*SLC6A3*), and rs4680 COMT (Val158Met) genes with the occurrence of MLIC in PD patients.

## 2 MATERIALS AND METHODS

### 2.1 Subjects

The inclusion of patients with idiopathic PD into the study was based on the clinical diagnosis of the disease according to the UK PDS Brain Bank criteria and the EFNS/MDS‐ES recommendations, and chronic levodopa therapy lasting at least 3 years. Seventy‐six patients have been included to the PD group and 60 to the control group. PD patients were treated as follows: (a) levodopa and ropinirole—33 patients (43.4%), (b) levodopa monotherapy—19 patients (25%), (c) levodopa and piribedil 75–150 mg daily (mean 109.36 mg daily)—eight patients (10.5%), (d) levodopa and ropinirole and entacapone—six patients (7.9%), (e) levodopa and ropinirole and amantadine—three patients (4%), (f) levodopa and piribedil (150 mg daily) and amantadine— 3 patients (4%), (g) levodopa and amantadine—two patients (2.6%), (h) levodopa and entacapone and amantadine—one patient (1.3%), (i) levodopa and ropinirole and entacapone and amantadine—one patient (1.3%). The levodopa equivalent dose (LED) was calculated (Tomlinson et al., 2010). Among 60 controls, 28 had mild ischemic stroke, 21—transient ischemic attack, four—posttraumatic subdural hematoma non requiring surgical intervention, two—discopathy, two—unruptured brain aneurysm, one—normal pressure hydrocephalus, one—epilepsy and one—tension‐type headache. The progression of PD was evaluated using Hoehn and Yahr scale, the motor symptoms severity—using the Unified Parkinson's Disease Rating Scale (UPDRS) part III (in ON phase) and complications of therapy—using UPDRS IV ([A] dyskinesias, [B] clinical fluctuations, and [C] other complications) or: (IV A ‐ dyskinesias, IV B ‐ clinical fluctuations, and IV C ‐ other complications). The study protocol was approved by the relevant local Bioethics Committee and conformed to the standard set by the Declaration of Helsinki. All participants gave their informed consent prior to their inclusion in the study.

The baseline characteristics of the groups were presented in Table 1. There was no difference in sex between the PD group and controls. PD patients were younger than controls, and men were younger than women in both PD and control group. There were no differences in PD duration, daily dose of levodopa and ropinirole, LED, duration of levodopa and ropinirole treatment, UPDRS III and Hoehn and Yahr scale scores between male and female in the PD group. Male PD patients were younger at disease onset than female. MLIC occurred in 43 (56.6%) PD patients (24 female, 19 male) with mean duration of 3.24 (range 0.5–8) years and after on average 6.16 years of dopaminergic treatment. There were 16 (37.2%) patients with only on‐off phenomena, and 27 (62.8%) with both on‐off phenomena and dyskinesias.

**Table 1:** Parkinson's disease patients and controls baseline characteristics

	PD patients	Control group	*p* value PD versus controls
Number of patients	76	60	–
Sex			
Female	40	29	ns^a^
Male	36	31	
Age (years) mean ± standard deviation (range)			
All	65.7 ± 9.4 (41–79)	70.5 ± 12.0 (51–88)	.01
Female	68.4 ± 9.0	73.6 ± 10.1	
Male	62.6 ± 8.9	66.9 ± 12.5	
*p* value Female versus Male	<.01	<.01	
Age at PD onset (years) mean** **±** **standard deviation (range)			
All	55.4 ± 8.9 (32–72)		
Female	57.6 ± 8.3		
Male	52.9 ± 8.9		
*p* value Female versus Male	<.05		
Duration of PD (years) mean** **±** **standard deviation (range)	10.3 ± 4.6 (3–23)		
Dose of levodopa (mg/daily) mean ± standard deviation (range)	812.5 ± 381.6 (200–2,550)		
Duration of levodopa treatment (years) mean ± standard deviation (range)	8.6 ± 4.4 (1–20)		
Dose of ropinirole (mg/daily) mean ± standard deviation (range)	3.3 ± 3.4 (0–12)		
Duration of ropinirole treatment (years) mean ± standard deviation (range)	2.4 ± 2.8 (0–12)		
Levodopa equivalent dose (mg/daily) mean ± standard deviation (range)	871.8 ± 385.3 (200–2,550)		
UPDRS III score mean ± standard deviation (range)	18.9 ± 9.6 (3–43)		
Hoehn and Yahr scale score mean ± standard deviation (range)	2.7 ± 0.7 (1.5–4.0)		

Abbreviations: ns, nonsignificant; PD, Parkinson's disease; UPDRS, Unified Parkinson's Disease Rating Scale.

a Fisher's exact test, 2 × 2 contingency table, nothing—Student's *t* test.

### 2.2 Genetic analysis

Genomic DNA was isolated from peripheral blood samples using GeneJET blood genomic DNA purification kit (Thermo Fisher Scientific) following the manufacture's recommended protocol. DNA concentrations were determined using the NanoDrop 2000 UV/VIS spectrophotometer (Thermo Fisher Scientific). Allelic discrimination of the rs6265 BDNF (Val66Met), rs397595 DAT (*SLC6A3*), and rs4680 COMT (Val158Met) gene variants were genotyped by using pre‐validated allelic discrimination TaqMan Real‐Time PCR Assay (Applied Biosystems).

### 2.3 Statistical analysis

Statistical analysis was performed using Student's *t* test, Mann–Whitney test, Fisher's exact test, and contingency table chi‐square followed by Yates correction test (Statistica v. 10, StatSoft, Inc., http://www.statsoft.com). The chi‐square test was used to analyze frequencies of the genotypes for deviations from Hardy–Weinberg equilibrium (HWE) in PD patients, controls, and PD patients with and without MLIC. Testing deviation from the HWE, Pearson's chi‐squared test, or Fisher's exact test was used to observed genotype frequencies obtained from the data and the expected genotype frequencies obtained using the HWE. Odds ratios (OR) and 95% confidence intervals (95% CI) were calculated using multinominal logistic regression (SPSS v.13). The differences were considered significant if the *p* value was >.05. Multivariate projection methods for data exploration—orthogonal partial least squares (OPLS) analysis and OPLS discriminant analysis (OPLS‐DA)—were used to analyze qualitative genetic data. The quality of OPLS/OPLS‐DA model was described by two parameters: goodness of fit (*R* ^2^cum) and goodness of prediction reported as the cumulative score across all of the components (*Q* ^2^cum). Model was considered as good when *R* ^2^cum and *Q* ^2^cum values were equal or greater than 0.5. The variable importance in the projection (VIP) value for model was calculated to indicate their contribution to the classification. Variables with VIP value >1.0 were considered significantly different. The model was validated using the analysis of variance testing of cross‐validated predictive residuals (CV‐ANOVA). Multivariate analysis (OPLS/OPLS‐DA) was performed using the SIMCA‐P software package (Version 14, Umetrics AB).

## 3 RESULTS

### 3.1 Genotype distributions of rs6265 BDNF, rs397595 DAT, and rs4680 COMT polymorphisms in PD patients and controls

Genotype distributions of BDNF, DAT, and COMT polymorphisms with OR and 95% CI in PD patients and controls have been shown in Table 2. Both groups were in HWE concerning DAT and COMT genotypes (*p* > .05). The distribution of BDNF genotypes did not show significant deviation from HWE in the controls (*p* > .05); however, BDNF polymorphism did not display HWE in the PD group (*p* < .0001). A higher frequency of AG BDNF genotype was seen in PD patients (67.1%) and of GG BDNF genotype in controls (73.3%). Sex did not have influence on distribution of these genotypes both in PD patients and controls. The multinominal logistic regression analysis showed significant differences in the distribution of BDNF genotypes (*p* < .0001) and no differences in distributions of DAT and COMT genotypes between PD patients and controls. The risk of PD in subjects with the AG BDNF genotype was increased sixfold (OR = 6.12, 95% CI = 2.88–13.02, *p* < .0001). No such association was observed with GG and AA homozygotes, although the low frequency of AA genotype and small sample size should be noted.

**Table 2:** Genotype frequencies of rs6265 BDNF (Val66Met), rs397595 DAT (*SLC6A3*) and rs4680 COMT (Val158Met) polymorphisms in Parkinson's disease patients and controls

Gene		Genotypes	PD patients *n* (%)	Controls *n* (%)	*χ* ^2^ PD/controls	*p* value PD/controls	OR (95% CI)	*p* value PD versus controls
BDNF	All	GG	24 (31.6%)	44 (73.3%)	17.322/0.047	<.0001/.828	0.17 (0.08–0.36)	<.0001
		AG	51 (67.1%)	15 (25.0%)			6.12 (2.88–13.02)	<.0001
		AA	1 (1.3%)	1 (1.7%)			0.79 (0.05–12.84)	1.0
	Female	GG	14 (35.0%)	21 (72.4%)	9.273/0.183	.002/.669	0.21 (0.07–0.58)	.005
		AG	26 (65.0%)	7 (24.1%)			5.84 (2.0–17.02)	.002
		AA	0 (0%)	1 (3.5%)			0	–
	Male	GG	10 (27.8%)	23 (74.2%)	8.346/0.680	.004/.410	0.13 (0.05–0.40)	.0004
		AG	25 (69.4%)	8 (25.8%)			6.53 (2.23–19.1)	.0009
		AA	1 (2.8%)	0 (0%)			0	–
DAT	All	AA	38 (50.0%)	39 (65.0%)	3.726/0.234	.054/.628	0.54 (0.27–1.08)	.11
		AG	36 (47.4%)	18 (30.0%)			2.1 (1.03–4.28)	.04
		GG	2 (2.6%)	3 (5.0%)			0.51 (0.08–3.18)	.65
	Female	AA	20 (50.0%)	20 (69.0%)	2.0575/3.961	.151/.047	0.45 (0.17–1.23)	.18
		AG	19 (47.5%)	6 (20.7%)			3.47 (1.16–10.34)	.04
		GG	1 (2.5%)	3 (10.3%)			0.22 (0.02–2.25)	.3
	Male	AA	18 (50.0%)	19 (61.3%)	1.672/1.786	.196/.181	0.63 (0.24–1.67)	.5
		AG	17 (47.2%)	12 (38.7%)			1.42 (0.53–3.76)	.65
		GG	1 (2.8%)	0 (0%)			–	–
COMT	All	AA	28 (36.8%)	21 (35.0%)	1.599/1.769	.206/.183	1.08 (0.53–2.2)	1.0
		AG	34 (44.7%)	28 (46.7%)			0.93 (0.47–1.83)	1.0
		GG	14 (18.4%)	11 (18.3%)			1.0 (0.42–2.41)	.8
	Female	AA	20 (50.0%)	20 (69.0%)	2.0575/3.961	.151/.047	0.45 (0.17–1.23)	.18
		AG	19 (47.5%)	6 (20.7%)			3.47 (1.16–10.34)	.04
		GG	1 (2.5%)	3 (10.3%)			0.22 (0.02–2.25)	.3
	Male	AA	18 (50.0%)	8 (25.8%)	0.252/0.034	.616/.853	2.88 (1.02–8.1)	.04
		AG	14 (38.9%)	16 (51.6%)			0.6 (0.23–1.58)	.4
		GG	4 (11.1%)	7 (22.6%)			0.43 (0.11–1.63)	.4

Abbreviations: 95% CI, 95% confidence interval; BDNF, brain‐derived neurotrophic factor; COMT, catechol‐O‐methyltransferase; DAT, dopamine transporter; OR, odds ratio; PD, Parkinson's disease; *χ* ^2^, chi‐square.

### 3.2 OPLS‐DA of genotypes in PD patients and controls

**Figure 1:**
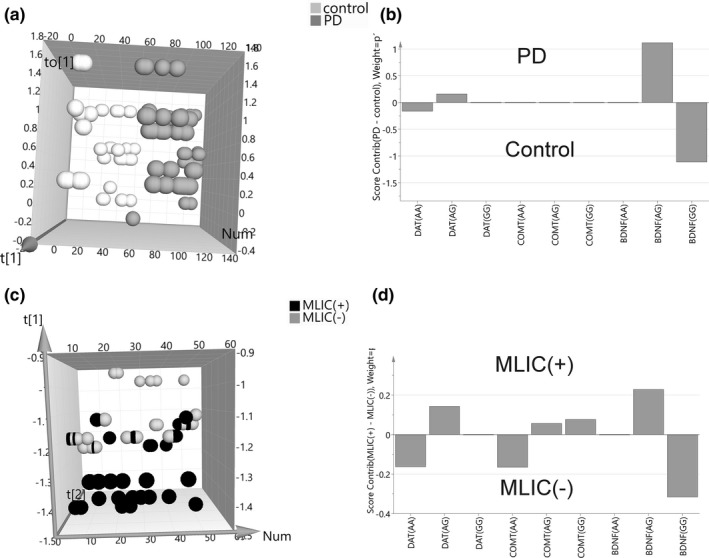
OPLS/OPLS‐DA analysis of rs6265 BDNF (Val66Met), rs397595 DAT (SLC6A3), and rs4680 COMT (Val158Met) polymorphisms in Parkinson's disease patients versus controls (a, b) and in Parkinson's disease patients with versus without motor levodopa‐induced complications (c, d). (a) Orthogonal partial least squares‐discriminant analysis (OPLS‐DA) scores plot of Parkinson's disease (PD) patients and control genetic data showing separation between both groups. The score vector to[1] represents within‐group variation in the orthogonal component, while the score vector t[1] represents between‐group variation in the predictive component, Num—number of sample. (b) score contribution of genetic data to group differentiation. (c) orthogonal partial least squares‐discriminant analysis (OPLS) scores plot of two Parkinson's disease (PD) groups (MLIC(+)—with motor levodopa‐induced complications and MLIC(−)—without motor levodopa‐induced complications) genetic data showing separation between both groups. The score vector t[1] represents first component, while the score vector t[2] represents second component, Num—number of sample. (d) score contribution of genetic data to group differentiation. BDNF, brain‐derived neurotrophic factor; COMT, catechol‐O‐methyltransferase; DAT, dopamine transporter

Orthogonal partial least squares‐discriminant analysis indicated that it was possible to differentiate PD patients and controls based on genetic data. Model consisted of one predictive component, two orthogonal components, and one component unrelated to genes and groups. The model was described by cumulative variation (*R* ^2^cum = 0.776, *Q* ^2^cum = 0.111) and was validated using CV‐ANOVA (*p* = .02). The most important genotypes involved in groups differentiation were as follows: AG BDNF, GG BDNF, and AA DAT (VIP > 1). AG BDNF and AG DAT were correlated with PD while GG BDNF and AA DAT with controls (Figure 1a,b).

### 3.3 Clinical comparison between Parkinson's disease patients with and without MLIC

Clinical differences between PD patients with and without MLIC were presented in Table 2. Patients with MLIC were younger, developed PD earlier and had more advanced PD stage in Hoehn and Yahr scale in comparison with patients without MLIC. Those differences in age and age at disease onset were observed in male but not in female PD patients, whereas higher score in Hoehn and Yahr scale affected female but not male PD patients. Patients with MLIC (female and male) took higher daily levodopa dose and LED and were treated with levodopa longer in comparison with patients without MLIC. There was no difference in sex, mean duration of PD, daily dose of ropinirole, duration of ropinirole treatment, and UPDRS III and IVC score between those groups. Statistical analysis indicated no clinical differences between patients with only on‐off phenomena and patients with both on‐off phenomena and dyskinesias (except for scores in UPDRS IV A and B rating dyskinesias and clinical fluctuations; Table 3).

**Table 3:** Clinical differences between Parkinson's disease patients with and without motor levodopa‐induced complications

	PD patients with MLIC				PD patients without MLIC	*p* value PD patients with MLIC versus without MLIC
	All	With only on‐off	With both on‐off and dyskinesias	*p* value PD patients with only on‐off versus with both on‐off and dyskinesias		
Number (percentage) of patients	43 (56.6%)	16 (37.2%)	27 (60.4%)	–	33 (43.4%)	–
Sex						
Females number (percentage)	24 (55.8%)	8 (50%)	16 (59.3%)	ns^a^	16 (44.2%)	ns^a^
Males number (percentage)	19 (44.2%)	8 (50%)	11 (40.7%)		17 (55.8%)	
Age (years) mean ± standard deviation (range)						
All	63.4** **±** **9.2 (41–79)	61.8** **±** **7.8	64** **±** **10	ns	68.6** **±** **8.8 (51–88)	.017
Female	66.4 ± 10.4	60.9** **±** **9.4	67.3** **±** **8.9	ns	67.9 ± 8.8	ns
Male	60.9 ± 8.4	62.6** **±** **6.3	59.3** **±** **10.0	ns	68.5 ± 8.3	.011
*p* value Female versus Male	ns	ns	ns		ns	
Age at PD onset (years) mean ± standard deviation (range)						
All	52.7** **±** **8.3 (32–69)	51.9** **±** **7.9	53.0** **±** **8.6	ns	58.8** **±** **8.5 (39–72)	.003
Female	54.2 ± 9.6	51.6** **±** **10.8	55.5** **±** **7.8	ns	58.8 ± 7.3	ns
Male	50.0 ± 7.5	52.3** **±** **3.9	49.3** **±** **8.8	ns	59.5 ± 8.1	.001
*p* value Female versus Male	ns	ns	ns		ns	
Duration of PD (years) mean ± standard deviation (range)	10.7** **±** **4.1 (4–20)	9.8** **±** **3.9 (4–18)	11.0** **±** **4.2 (5–20)	ns	9.8** **±** **5.1 (3–23)	ns
Dose of levodopa (mg/daily) mean ± standard deviation (range)	944.2** **±** **406.2 (200–2,550)	932.2** **±** **262.5 (600–1,455)	975.0** **±** **475.8 (200–2,550)	ns	640.9** **±** **266.5 (200–1,250)	<.001
Duration of levodopa treatment (years) mean ± standard deviation (range)	9.4** **±** **4.2 (1–20)	8.4** **±** **4.0 (3–16)	9.7** **±** **4.3 (1–20)	ns	7.6** **±** **4.3 (1–20)	.025
Dose of ropinirole (mg/daily) mean ± standard deviation (range)	3.3** **±** **3.4 (0–12)	3.6** **±** **3.7 (0–12)	3.3** **±** **3.4 (0–9)	ns	3.2** **±** **3.3 (0–10)	ns
Duration of ropinirole treatment (years) mean ± standard deviation (range)	2.1** **±** **2.4 (0–10)	2.2** **±** **2.5 (0–8)	2.3** **±** **2.6 (0–10)	ns	2.7** **±** **3.3 (0–12)	ns
Levodopa equivalent dose (mg/daily) mean ± standard deviation (range)	1,008.0** **±** **395.6 (200–2,550)	932.2** **±** **262.5 (600–1,455)	965.4** **±** **476.5 (200–2,250)	ns	694.3** **±** **291.9 (215–1,360)	<.001
UPDRS III score mean ± standard deviation (range)	18.4** **±** **9.5 (4–40)	19.4** **±** **10.8 (0–35)	16.9** **±** **8.8 (4–38)	ns	19.7** **±** **9.8 (3–43)	ns
UPDRS IV A score mean ± standard deviation (range)	2.3** **±** **2.4 (0–9)	0	3.5** **±** **2.2 (1–9)	<.001	0	<.001
UPDRS IV B score mean ± standard deviation (range)	3.2** **±** **1.0 (1–5)	2.5** **±** **1.1 (0–4)	3.4** **±** **0.9 (2–5)	.012	0	<.001
UPDRS IV C score mean ± standard deviation (range)	0.6** **±** **0.8 (0–3)	0.5** **±** **0.7 (0–2)	0.6** **±** **0.8 (0–3)	ns	0.3** **±** **0.6 (0–2)	ns
Hoehn and Yahr scale mean ± standard deviation (range)						
All	2.9** **±** **0.7 (1.5–4)	2.7** **±** **0.6 (2–4)	2.8** **±** **0.6 (1.5–4)	ns	2.5** **±** **0.6 (1.5–4)	.041
Female	3.1 ± 0.6	2.6** **±** **0.5	3.0** **±** **0.7	ns	2.5 ± 0.7	.005
Male	2.6 ± 0.6	2.8** **±** **0.7	2.6** **±** **0.5	ns	2.4 ± 0.5	ns
*p* value Female versus Male	.009	ns	ns		ns	

Abbreviations: MLIC, motor levodopa‐induced complications; ns, nonsignificant; PD, Parkinson's disease; UPDRS, Unified Parkinson's Disease Rating Scale.

a Fisher's exact test, 2 × 2 contingency table, nothing—Mann–Whitney test.

### 3.4 Genotype distributions and OPLS analysis of rs6265 BDNF, rs397595 DAT, and rs4680 COMT polymorphisms in PD patients with and without MLIC

Genotype distributions of BDNF, DAT, and COMT polymorphisms with OR and 95% CI in PD patients with and without MLIC were shown in Table 4. The frequencies of DAT and COMT genotypes displayed HWE in both PD groups (*p* > .05) while the distribution of BDNF genotypes showed significant deviation from HWE also in both PD groups (*p* = .001 and *p* = .009, respectively) (Table 4) and in MLIC subgroups (patients with only on‐off phenomena and patients with both on‐off phenomena and dyskinesias: *p* = .016 and *p* = .018, respectively) as well. The multinominal logistic regression analysis showed no differences in distributions of BDNF, DAT, and COMT genotypes between PD groups without and with MLIC as well as between MLIC subgroups (*p* > .05), and sex did not influence those distributions. OPLS analysis demonstrated that it was possible to differentiate PD groups with and without MLIC upon gene analysis. Model consisted of two predictive components and one orthogonal component. The model was described by cumulative variation (*R* ^2^cum = 0.834, *Q* ^2^cum = 0.678) and was validated using CV‐ANOVA (*p* < .0001). The most important genotypes involved in groups differentiation were as follows: AG BDNF, AG DAT, and AA DAT (VIP > 1). Genotype combination of AG BDNF, AG DAT, and GG COMT was correlated with PD patients with MLIC and genotype combination of GG BDNF, AA DAT, and AA COMT with PD patients without MLIC (Figure 1c,d).

**Table 4:** Genotype frequencies of rs6265 BDNF (Val66Met), rs397595 DAT (SLC6A3) and rs4680 COMT (Val158Met) polymorphisms in Parkinson's disease patients with and without motor levodopa‐induced complications

Gene		Genotypes	PD patients with MLIC *n* (%)	PD patients without MLIC *n* (%)	*χ* ^2^ with/without MLIC	*p* value with/without MLIC	OR (95% CI)	*p* value with versus without MLIC
BDNF	All	GG	11 (26.2%)	13 (38.2%)	11.115/6.788	.001/.009	0.57 (0.22–1.52)	.4
		AG	30 (71.4%)	21 (61.8%)			1.55 (0.59–4.05)	.5
		AA	1 (2.4%)	0 (%)			–	
	Female	GG	7 (29.2%)	7 (43.8%)	7.218/2.450	.007/.118	0.53 (0.13–1.99)	.5
		AG	17 (70.8%)	9 (56.2%)			1.89 (0.5–7.09)	.5
		AA	0 (%)	0 (0%)			–	
	Male	GG	4 (22.2%)	6 (33.3%)	4.247/4.500	.039/.034	0.57 (0.13–2.51)	.7
		AG	13 (72.2%)	12 (66.7%)			1.3 (0.31–5.39)	1.0
		AA	1 (5.6%)	0 (0%)			–	
DAT	All	AA	18 (42.9%)	20 (58.8%)	2.135/2.2853	.144/.131	0.53 (0.21–1.31)	.3
		AG	22 (52.3%)	14 (41.2%)			1.57 (0.63–3.91)	.5
		GG	2 (4.8%)	0 (0%)			–	
	Female	AA	11 (45.8%)	9 (56.3%)	1.059/1.2544	.303/.263	0.66 (0.18–2.35)	.8
		AG	12 (50.0%)	7 (43.8%)			1.29 (0.36–4.58)	1.0
		GG	1 (4.2%)	0 (0%)			–	
	Male	AA	7 (38.9%)	11 (61.1%)	1.125/1.0488	.289/.306	0.41 (0.11–1.55)	.3
		AG	10 (55.6%)	7 (38.9%)			1.96 (0.52–7.41)	.5
		GG	1 (5.5%)	0 (0%)			–	
COMT	All	AA	15 (35.7%)	13 (38.2%)	0.656/0.001	.418/.982	0.9 (0.35–2.29)	1.0
		AG	18 (42.9%)	16 (47.1%)			0.84 (0.34–2.09)	.9
		GG	9 (21.4%)	5 (17.7%)			1.58 (0.48–5.26)	.6
	Female	AA	5 (20.8%)	5 (31.3%)	0.174/0.237	.676/.627	0.58 (0.14–2.46)	.7
		AG	13 (54.2%)	7 (43.7%)			1.52 (0.43–5.43)	.8
		GG	6 (25.0%)	4 (25.0%)			1 (0.23–4.31)	1.0
	Male	AA	10 (55.5%)	8 (44.4%)	2.148/0.572	.143/.450	1.56 (0.42–5.82)	.7
		AG	5 (27.8%)	9 (50.0%)			0.38 (0.1–1.54)	.3
		GG	3 (16.7%)	1 (5.6%)			3.4 (0.32–36.27)	.6

Abbreviations: 95% CI, 95% confidence interval; BDNF, brain‐derived neurotrophic factor; COMT, catechol‐O‐methyltransferase; DAT, dopamine transporter; MLIC, motor levodopa‐induced complications; OR, odds ratio; PD, Parkinson's disease; *χ* ^2^, chi‐square.

## 4 DISCUSSION

From years, BDNF gene has been considered to be a potential candidate for susceptibility to PD due to decreased BDNF mRNA expression and protein content observed in substantia nigra of PD patients (Mogi et al., 1999). In the present study conducted on Polish Caucasians subjects, PD patients were significantly more frequently carriers of the heterozygous AG allele of BDNF Val66Met polymorphism and controls were significantly more frequently carriers of the homozygous GG allele. The multinominal logistic regression analysis demonstrated the sixfold increased risk of PD in carriers of the AG BDNF genotype. No such association was observed for AA homozygotes probably due to the low frequency of homozygous genotype and small samples size. The presence of the allele A led to lower activity dependent secretion of the protein; therefore, heterozygotes AG also showed impaired intracellular distribution and lower secretion levels of BDNF, even though presumably not as pronounced as homozygotes AA (Egan et al., 2003). The association between BDNF Val66Met polymorphism and susceptibility to PD was previously investigated with inconsistent results. There was no association found between that SNP and the risk of developing PD independently of the ethnicity in several meta‐analysis (Dai et al., 2013; Mariani et al., 2015) except for one that identified an association between that SNP and PD in Europeans but not in Asians (Lee & Song, 2014). Other SNPs of the BDNF gene were investigated, and a relationship between BDNF 712A/G AG/AA genotypes and PD was suggested in Chinese Han population (Chen et al., 2011). Our data support the hypothesis that the insufficient supply of BDNF may contribute to the neurodegenerative process in PD.

We did not evidence associations between individual DAT (rs397595) and COMT Val158Met polymorphisms and PD but OPLS‐DA model showed that genotype combination of AG BDNF and AG DAT was correlated with PD patients and genotype combination of GG BDNF and AA DAT with controls. The link between DAT polymorphisms and susceptibility to PD might consist in changes in DAT expression and synaptic uptake of potential neurotoxins responsible for nigral cell death (Tipton & Singer, 1993). Among Japanese PD patients, no evidence of association between the 1215A/G polymorphism in DAT gene and PD was found in one study (Kimura, Matsushita, Arai, Takeda, & Higuchi, 2001), while such association was confirmed in two others (Morino et al., 2000; Nishimura, Kaji, Ohta, Mizuta, & Kuno, 2002) and the 10‐copy genotype of 40‐pb VNTR polymorphism in that gene was suggested to make a protective factor for the development of PD in male Taiwanese (Lin et al., 2003). Genetic variations in COMT gene were proposed as a susceptibility factor for PD because of their role in the inactivation of many biologically active and toxic catechols. The allele A of the COMT Val158Met polymorphism is linked to low (L) COMT activity of the enzyme, in contrast to the allele G linked to high (H) COMT activity (Lotta et al., 1995) and the COMT enzymatic activity in women is lower than in men (Xie, Ho, & Ramsden, 1999). The involvement of COMT Val158Met SNP in the susceptibility of PD was detected in Asian populations (Chuan et al., 2015; Kiyohara et al., 2011) and of COMT Val108Met SNP (L/L genotype) in younger subjects and in women among US patients with both parents of European origin (Goudreau et al., 2002). Meta‐analysis showed both no association between COMT Val108/158Met SNP and PD regardless of ethnicity (Jiménez‐Jiménez, Alonso‐Navarro, García‐Martín, & Agúndez, 2014) and association of COMT Val158Met PSN and the risk of PD in Asians rather than Caucasians (Lechun, Yu, Pengling, & Canggi, 2013). That risk was found with the COMT H/L genotype of Val108Met SNP in interaction with the MAO‐B G genotype of polymorphism of intron 13 in female PD patients in Polish population (Białecka et al., 2005). The ethnicity remains clearly a key factor for genetic susceptibilities in PD and considering not only single polymorphisms but also overlapping effects of more than one seems to be appropriate.

Motor levodopa‐induced complications (there were in the majority patients with both on‐off phenomena and dyskinesias) occurred in 56.6% of PD patients (55.8% females) and were associated with younger age and younger age at PD onset (in men), more increased disease severity (in women), higher daily levodopa dose and LED and longer duration of levodopa therapy. These findings except for sex differences (probably due to low sample size) are coherent with results of previous studies on clinical risk factors of MLIC (Olanow et al., 2013; Scott, Macleod, & Counsell, 2016). No association was found between individual BDNF, DAT, and COMT polymorphisms and MLIC in multinominal logistic regression analysis; meanwhile, genotypes combination of AG BDNF, AG DAT, and GG COMT was correlated with MLIC and genotypes combination of GG BDNF, AA DAT, and AA COMT with lack of MLIC in PD patients in OPLS analysis. Our results on individual BDNF Val66Met and COMT Val158Met polymorphisms are in concordance with studies on Israeli and Australian/UK PD patients which showed no association between those SNPs and prevalence or time to onset of levodopa‐induced dyskinesias (Cheshire et al., 2014; Kaplan et al., 2014). However, in a single study on PD patients in the majority Caucasians, the allele A of the BDNF Val66Met SNP (related to decreased protein secretion) was associated with higher risk of developing levodopa‐induced dyskinesias (Foltynie et al., 2009), and futhremore the low‐activity allele A of the COMT Val158Met SNP was reported to increase the risk of levodopa‐induced dyskinesias in Dutch PD patients (de Lau et al., 2012), and to decrease the risk of wearing‐off phenomena in Chinese PD patients (Wu et al., 2014). The 9‐copy allele of 40‐pb VNTR polymorphism in DAT gene was found to be a predictor for the occurrence of not only dyskinesias but also psychosis in Caucasians (Kaiser et al., 2003), while the allele C of the rs393795 SNP in that gene was reported to extend time to dyskinesias onset in Israeli (Kaplan et al., 2014) levodopa‐treated PD patients. No associations were found between combined BDNF Val66Met, COMT Val158Met, and T941G MAO‐A SNPs and the prevalence or time to onset of levodopa‐induced dyskinesias in Australian/UK PD patients (Cheshire et al., 2014). Combination of BDNF, DAT, and COMT polymorphisms and the occurrence of MLIC were investigated for the first time.

The effect of BDNF on motor function is suggested by the association between the Val66Met SNP and reduced experience‐dependent plasticity of motor cortex (Kleim et al., 2006) as well as impaired synaptic plasticity in the nigrostriatal system (Egan et al., 2003) which could play a role in the development of MLIC in PD patients. Lower DAT levels in nigrostriatal neurons are associated with increased DA turnover and greater oscillations in synaptic DA concentrations in PD (Sossi et al., 2007). We hypothesize that allele G of rs397595 DAT polymorphism, possibly like allele A of another SNP—rs393795—in this gene (Kaplan et al., 2014), might be related to lower DAT level contributing to the development of MLIC. A potential link between polymorphisms of COMT gene and susceptibility to MLIC is proposed because of increased risk of MLIC in PD patients on higher cumulative levodopa dose (Scott et al., 2016) and also the probable relationship of high COMT activity genotype with faster levodopa catabolism and less stable serum and brain drug concentrations leading to higher optimal clinically levodopa daily dose (Bialecka et al., 2008; Lee, Lyoo, Ulamnen, Syvänen, & Rinne, 2001). It was also found that PD patients carrying alleles that induce both high COMT activity and high MAO‐A expression genotypes, received higher maximum and daily LED (Cheshire et al., 2014). The allele G of the COMT Val158Met SNP is related to high activity of the enzyme (Lotta et al., 1995). Our results suggest a synergic effect of low secretion BDNF, probably low‐level DAT and high COMT activity genotypes on the occurrence of MLIC. Precise mechanisms through which BDNF, DAT, and COMT polymorphisms may interact which each other and conduct to a phenotype with greater tendency to developing MLIC require further and wider investigations.

Although the sample in the present study is ethnically homogenous, a low number of enrolled subjects is a limitation. Larger sample sizes are required not only to confirm our results but also to distinguish groups with different types of MLIC that may have both common and distinct pathomechanisms.

In conclusion, the present study confirmed the association of BDNF Val66Met SNP with the risk of PD and showed a potential synergic effect of BDNF Val66Met, rs397595 DAT, and COMT Val158Met SNPs on the occurrence of MLIC. This potential genetic biomarker may help to predict which PD patients are more placed at risk of these long‐term adverse effects of levodopa therapy.

## CONFLICT OF INTEREST

The authors declare that they have no conflict of interest.

## Data Availability

The data that support the findings of this study are available from the corresponding author upon reasonable request.
